# Atkinsonella hypoxylon virus capsid structure highlights the diversity of capsid proteins among the Partitiviridae

**DOI:** 10.1099/jgv.0.002209

**Published:** 2026-01-16

**Authors:** Micol Venturi, Matthew Calthorpe-Byrne, Beate Aftret, Donna McNeale, Bernd H.A. Rehm, Frank Sainsbury

**Affiliations:** 1 Centre for Cell Factories and Biopolymers, Institute for Biomedicine and Glycomics, Griffith University, Nathan, QLD 4111, Australia; 2 Australian Research Council Centre of Excellence in Synthetic Biology, Sydney, Australia; 3 electron Bio-Imaging Centre, Diamond Light Source Ltd, Didcot, Oxfordshire OX11 0DE, UK

**Keywords:** mycovirus, partitivirus, picobirnavirus, transient expression, virus-like particle

## Abstract

*Atkinsonella hypoxylon* virus (AhV) is a fungi-infecting betapartitivirus and the typical member of the *Partitiviridae*, a family of persistent viruses that infect a broad range of organisms. Partitiviruses have been largely overlooked following their designation as cryptic viruses. However, evidence is accumulating that they play an important role in the ecology of their hosts. Since the capsid proteins of partitiviruses have been implicated in virus–host interactions, exploring their structural biology may give clues into the evolution, horizontal transmission and host adaptation of partitiviruses. The capsid of AhV shares the same organization of other partitiviruses with 60 dimeric capsid protein protomers arranged with *T*=1 icosahedral symmetry. The structure, determined by cryo-electron microscopy to 2.4 Å, shows that AhV has a unique iteration on the protrusion domain with an extensive network of hydrophobic interactions among equivalent interdigitating loops at the dimerization interface. AhV also shares a conserved helical core in the shell domain, which we extend to all genera of the recognized partitiviruses using protein structure prediction. The helical core appears to be a conserved element of the picobirnavirus lineage of capsid protein folds and provides a template onto which various elaborations of the protrusion domain have evolved. The involvement of the protrusion in virus–host interactions has previously been proposed, and our findings provide evidence of a structural device enabling capsid protein diversification during the evolution of the *Partitiviridae*.

## Data availability

*Atkinsonella hypoxylon* virus coat protein coordinates are deposited in the Protein Data Bank under accession code 8PHH. The cryo-electron microscopy reconstruction of the capsid is deposited in the EM Data Bank under accession code EMD-17662.

## Introduction

*Partitiviridae* is a family of persistent viruses, with species now identified from fungal, plant, protist, animal and bacterial hosts. The five genera recognized by the International Committee on the Taxonomy of Viruses [1] include the *Alphapartitivirus* and *Betapartitivirus* that infect both plants and fungi and *Deltapartitivirus*, *Gammapartitivirus* and *Cryspovirus* that are restricted to plant, fungal and protist hosts, respectively. However, cryspovirus-like partitiviruses (PVs) have since been found in yeast [2], and two proposed genera, *Epsilonpartitivirus* [3] and *Zetapartitivirus* [4,5], contain viruses that infect arthropods and fungi. Recently identified prokaryotic PVs form a distinct clade [6,8], leading to the recent establishment of a sister family, *Soropartitiviridae* [9]. There is an emerging recognition that PVs and PV-like viral entities are enormously successful. In addition to the regular discovery of new hosts, they may be the most abundant viruses in fungi [4] and wild plants [10].

Formerly known as cryptic viruses due to the apparent asymptomatic nature of PV infections, details are emerging of virus–host interactions that are beginning to shed light on the nature of their persistent infections. For example, a mutualistic relationship has been identified between pepper cryptic virus 1 and its plant host *Capsicum annuum* [11], and a conditionally mutualistic relationship may exist for white clover cryptic virus 1 and its leguminous host [12]. Conversely, several fungal PVs influence the virulence of their hosts [13,15], which may also be host-specific [16]. The majority of PVs have two genomic dsRNAs, each containing a single open reading frame. RNA-1 encodes an RNA-dependent RNA polymerase (RdRp) and RNA-2 encodes the capsid protein (CP). Despite such a limited repertoire of viral proteins, the determinants of virus–host interactions remain largely unknown. However, at least one alphapartitivirus CP modulates host defences [12] and structural features of the gammapartitiviruses and deltapartitiviruses have led to speculation that the CP plays a role [17,18].

The isometric capsid of PVs is composed of 60 quasi-symmetrical dimers arranged with *T*=1 icosahedral symmetry; however, there is considerable variability in the organization of CP domains between PV genera. Protrusions emanate from a conserved shell domain of deltapartitiviruses and gammapartitiviruses at distinctly different positions: at the CP dimerization interface in deltapartitiviruses forming an intertwined spike [17] and distal to the dimerization interface in gammapartitiviruses forming an arch [19,20]. High-resolution structures are only available for three virus capsids from these two genera and, as a result, the structural diversity within and between PV genera is not well understood. Greater understanding of PV capsid structures across the family may help elucidate a possible role of the CP in adapting to persistent lifestyles.

Here, we present the high-resolution capsid structure of the type species of the betapartitivirus genus, *Atkinsonella hypoxylon* virus (AhV). AhV possesses the typical bipartite genome of the *Partitiviridae* and was the first completely sequenced fungal virus of the family [21]. The genome segments and ORFs of the betapartitiviruses are the largest among the PVs [1], and the CP of AhV is 50%–58% longer than the structurally characterized gammapartitiviruses and deltapartitivirus. We find that the AhV CP model confirms another unique iteration on the core shell-forming design principle seen in the resolved PV CPs with a filled ‘butte’ structure characterized by extensive hydrophobic interactions between reciprocal penetrating loops at the dimer interface. Combined with *in silico* prediction of CP structures from recognized genera of the *Partitiviridae*, the findings highlight the flexibility of an evolutionarily conserved core CP structure to accommodate insertions and rearrangements to drive structural divergence.

## Methods

### Capsid expression and purification

The CP coding sequence from AhV RNA-2 (NC_003471.1) was ordered as a clonal gene with flanking *Age*I and *Xho*I restriction sites in pUC57 (https://www.geneuniversal.com/). Using these restriction sites, the CP sequence was subcloned into pEAQ-*HT* (GenBank accession GQ497234 [22]). Recombinant plasmid constructs were verified with Sanger sequencing by the Griffith University DNA Sequencing Facility. The resulting pEAQ-HT-AhV_CP expression vector was maintained in *Agrobacterium tumefaciens* strain LBA4404 transformed by electroporation and propagated at 28 °C in the presence of 50 µg ml^−1^ of kanamycin, 100 µg ml^−1^ streptomycin and 50 µg ml^−1^ rifampicin. LBA4404 cultures were resuspended in infiltration buffer [10 mM MES (pH 5.6) with 10 mM MgCl_2_ and 100 µM acetosyringone] to an OD_600_ of 0.4 and incubated for 2–4 h at ambient temperature. Leaves of 5-week-old *Nicotiana benthamiana* plants were vacuum infiltrated using a desiccator/vacuum chamber and incubated for 6–7 days. Approximately 25 g of infiltrated tissue was extracted in 50 ml extraction buffer [50 mM MOPS (pH 7.0) with 140 mM NaCl, 0.5 mM dithiothreitol added fresh] with complete EDTA-free protease inhibitor cocktail (https://www.sigmaaldrich.com/). The cell lysate was filtered using Miracloth to remove debris and centrifuged at 25,000 ***g*** for 25 min at 4 °C. A 50% iodixanol solution was prepared using a 6× PV buffer [120 mM MOPS (pH 7.0) with 840 mM NaCl], from which a cushion of 30% iodixanol in PV buffer [20 mM MOPS (pH 7.0) with 140 mM NaCl] was used to sediment AhV VLPs at 150,000 ***g*** for 3 h at 18 °C. The cushion containing the sample was collected and diluted in extraction buffer and then centrifuged at 150,000 ***g*** for 3 h at 18 °C. The pellet was resuspended with PV buffer in a small volume (100–200 µl) by vigorously pipetting up and down.

### Negative stain transmission electron microscopy

Capsid preparations (4 µl, ~0.25 mg ml^−1^) were applied to formvar/carbon-coated copper grids (ProSciTech) for 2 min. Grids were then washed on a droplet of water for 30 s two times and then stained on a droplet of Uranyless EM stain (ProSciTech) for 2 min. Excess stain was wicked away with filter paper and air-dried prior to storage. Images were taken on a Hitachi 7700 transmission electron microscope at 80 kV.

### Mass spectroscopy

Mass spectroscopy analysis was performed by the Australian Proteome Analysis Facility. In-gel trypsin digest was performed following reduction and alkylation of the sample resolved by SDS-PAGE. Peptides were subjected to LC-MS/MS analysis (Thermo, Ultimate 3000 HPLC coupled with Q-Exactive HFXMS), and the raw data file was processed using Proteome Discoverer (version 2.5.0.400, Thermo Scientific). The data was searched using search engines SequestHT specifying trypsin as the enzyme with a maximum of 2 missed cleavages, precursor mass tolerance of 20 ppm and fragment mass tolerance of 0.02 Da, allowing for dynamic modification of methionine (oxidation) and N-terminal acetylation and static cysteine carbamidomethylation. False discovery rates were set to <1% for peptide-spectrum matches and proteins using a custom database containing 77,048 *Nicotiana* sequences.

### Cryo-electron microscopy (cryo-EM) image processing

Image processing was carried out in RELION 4.0 [23]. Motion correction was performed with MOTIONCOR2 [24]. The contrast transfer function of motion corrected micrographs was determined with gCTF [25]. Approximately 110,000 particles were picked with the Laplacian picking tool in RELION. Particles were extracted in a 512×512 pixel box. The particle stack was reduced to 27,000 through iterative 2D and 3D classification. For 3D refinement, an initial model was generated from a small subset of this stack. 3D reconstruction resulted in a model of 3.0 Å. Further CTF refinement and Bayesian polishing resulted in a final 3D reconstruction with a resolution of 2.4 Å. Detailed parameters for data collection are shown in Table 1.

**Table 1.** Cryo-EM data collection, refinement and validation statistics

Data collection and processing	
Sample applications to grid	1
Magnification	81,000
Voltage (kV)	300
Electron exposure (e–/Å^2^)	50
Defocus range of micrographs (μm)	−0.5 to −2.0
Pixel size (Å)	0.829
Symmetry imposed	I
Initial particle images (no.)	110,000
Final particle images (no.)	27, 000
Map resolution (Å)	2.4
Fourier shell correlation threshold	0.143
Number of frames	50
**Refinement**	
Map sharpening *B* factor (Å^2^)	−94.4
Model composition	
Protein residues	1,027
Nucleic acids	0
R.m.s. deviations	
Bond lengths (Å)	0.003
Bond angles (°)	0.668
Validation	
Clashscore	14.73
Poor rotamers (%)	5.07
Ramachandran plot	
Favoured (%)	96.32
Allowed (%)	3.59
Disallowed (%)	0.09

The initial atomic model for AhV CP was built using ModelAngelo [26] and manually modified with COOT [27]. Refinement was carried out with Phenix real space refine [28], and the atomic model was validated using MolProbity [29]. All figures depicting AhV structures were prepared with UCSF Chimera and UCSF ChimeraX [30]. AhV CP coordinates are deposited in the Protein Data Bank (8PHH), and the cryo-EM reconstruction of the capsid is deposited in the EM Data Bank (EMD-17662).

### Phylogenetic analysis and *in silico* structural comparisons

Sequence alignments were made using the MAFFT plugin and the L-INS-i algorithm in Geneious Prime 2019.2.3 (https://www.geneious.com/). A maximum likelihood tree was built using IQ-TREE 2.3.5 [31] and automated model selection using ModelFinder [32] provided by Galaxy Australia [33]. Columns where at least 40% of the sequences had gaps were masked for tree building, and branch support was determined using UFBoot2 [34]. Additional cryspovirus CP sequences were identified by position-specific iterated blast (PSI-blast; https://blast.ncbi.nlm.nih.gov/Blast.cgi) with *Cryptosporidium parvum* virus 1 CP (YP_009508066.1) as the query sequence. Additional deltapartitivirus sequences were selected to represent the diversity within this genus from previously identified long and short form CP sequences [17].

The structures of 43 of 45 classified PV CPs and 19 additional CPs from putative deltapartitiviruses and cryspoviruses were predicted using AlphaFold (version 2.1.2), also via Galaxy Australia. The highest-ranking model was selected for further analysis in all cases. Structural alignments and molecular visualization were performed using UCSF ChimeraX [30].

## Results and discussion

### Transient plant-based expression of AhV coat proteins yields assembled virus-like particles

**Fig. 1.**
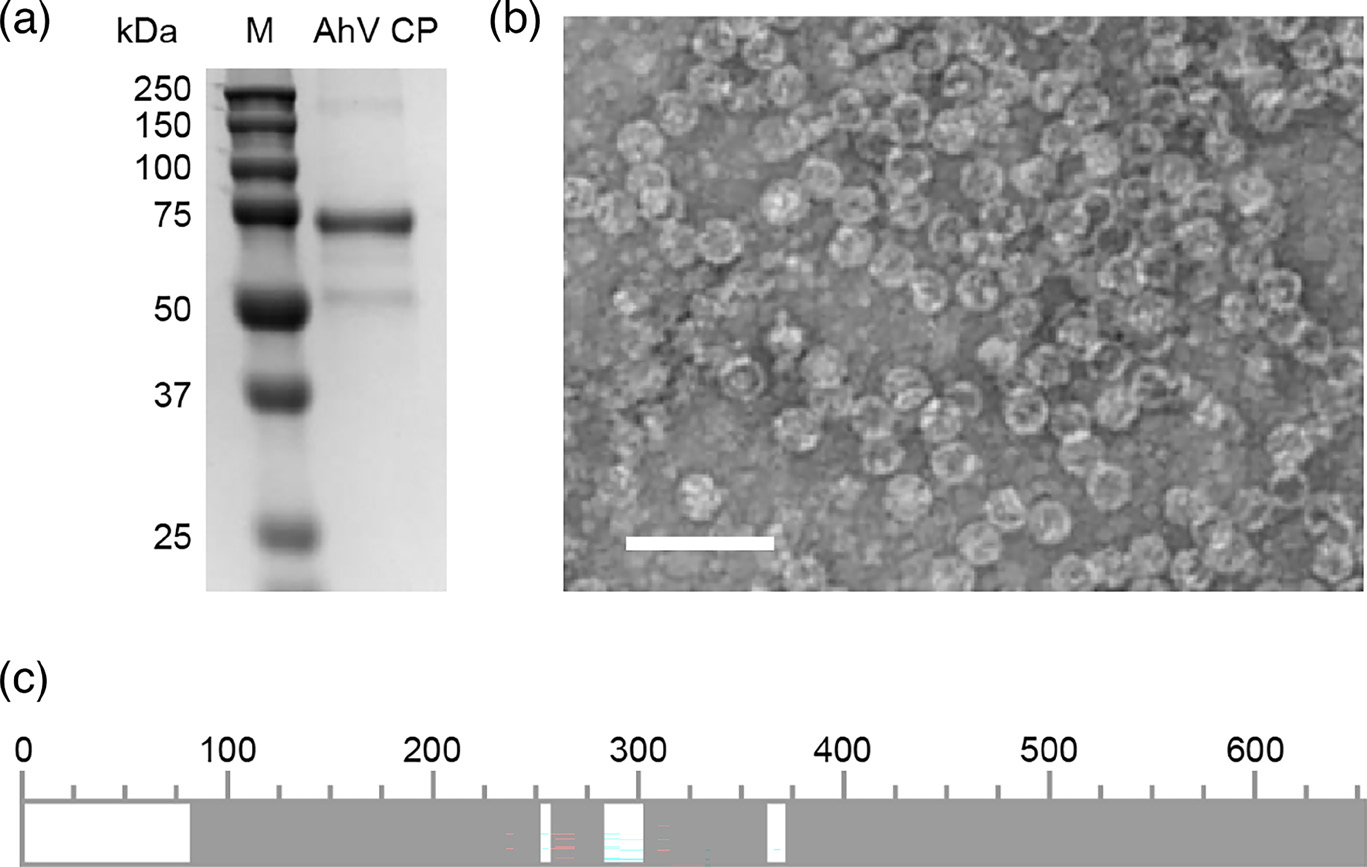
Expression and purification of AhV capsids. (**a**) SDS-PAGE gel of purified AhV showing the expected AhV CP band at ~74 kDa. The minor band at ~55 kDa is the large subunit of Rubisco. (**b**) Electron micrograph of purified AhV capsids. Bar=100 nm. (**c**) Schematic representation of peptide mass fingerprinting showing the coverage (grey) of identified peptides following tryptic digest of the ~74 kDa band in (**a**). Numbering refers to the AhV CP amino acid position and the sequence coverage is shown in detail in Fig. S1.

The 652 amino acid coding sequence for AhV CP was inserted into the pEAQ-*HT* expression vector [22]. Following infiltration of *A. tumefaciens* into leaves of mature *N. benthamiana* plants, the expression from this vector is driven by the cauliflower mosaic virus 35S promoter and engineered cowpea mosaic virus RNA-2 untranslated regions, supported by co-expression of the P19 suppressor of post-transcriptional gene silencing from tomato bushy stunt virus. Clarified lysates of homogenized leaf tissue were applied to an iodixanol cushion to isolate AhV capsids from host cell proteins, followed by centrifugal sedimentation to remove excess iodixanol and concentrate the capsids. SDS-PAGE showed a major band at the expected ~74 kDa (Fig. 1a), and the identity of this protein was confirmed as the AhV CP by mass spectroscopy of tryptic fragments (Figs 1c and S1, available in the online Supplementary Material). Despite the apparent full-length protein on the gel, no peptides were identified from the N-terminal 80 amino acids. We attribute this to the suitability of trypsin to digest the CP, although proteolytic modifications to the N-terminus during expression cannot be ruled out. The assembly of AhV CP into virus-like particles ~35 nm in diameter was confirmed by negative stain electron microscopy (Fig. 1b). These results show that transient expression in plants is a viable approach to the recombinant expression of fungal virus-like particles. Although similar outcomes have been found for various eukaryotic viruses such as those of plants [35,36], insects [37], fish [38] and mammals [39], it is notable that here we have not optimized the sequence for cross-kingdom heterologous expression. In addition, we have used a purification protocol for recombinant virus capsids that isolates assembled particles from host cell components in the first step, allowing simultaneous purification, buffer exchange and concentration with a second high-speed pelleting step. The resulting capsid preparations are, as we show below, suitable for high-resolution structure determination.

### Determination of the AhV capsid structure reveals a unique CP domain architecture

**Fig. 2.**
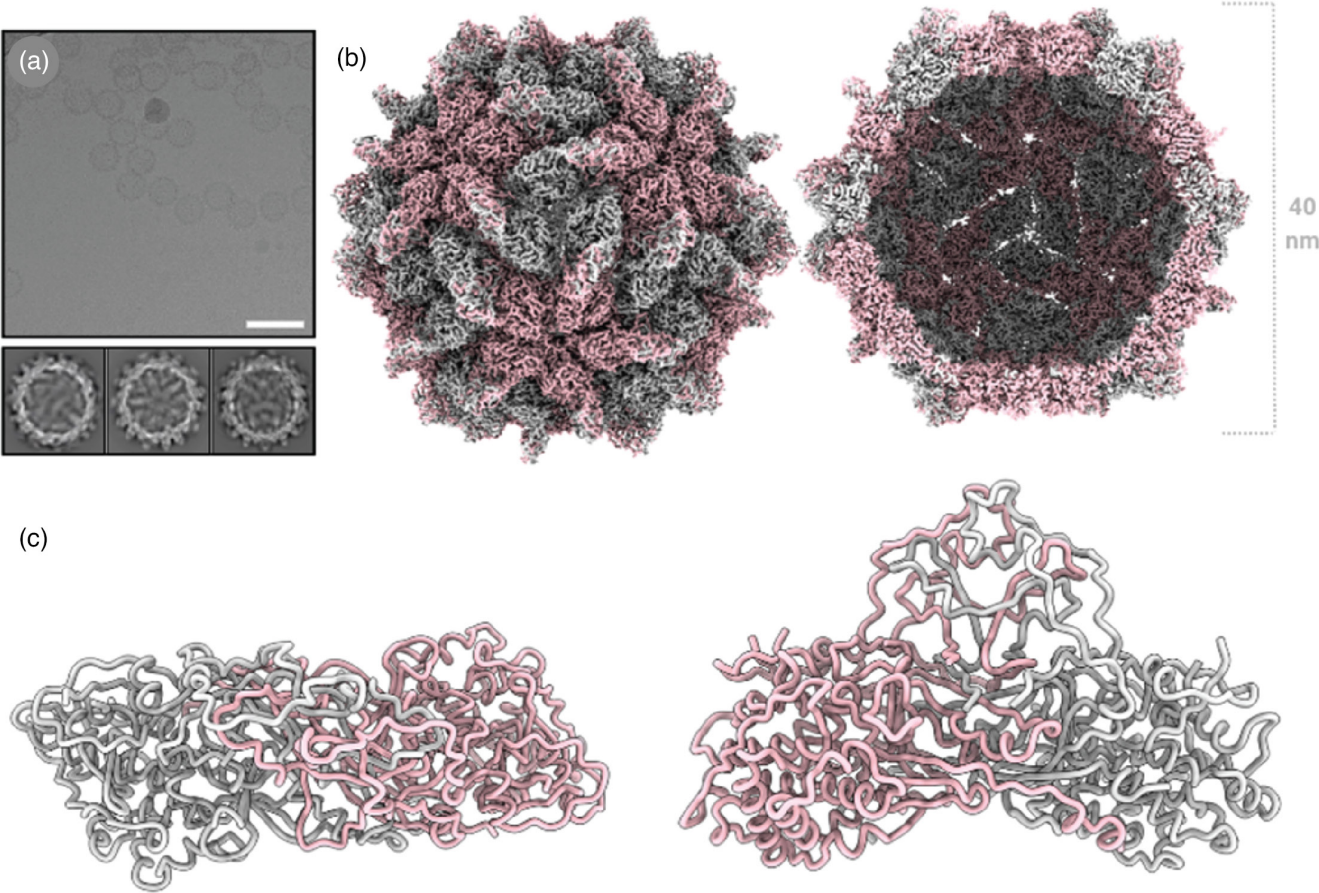
AhV capsid structure. (**a**) A section of a representative micrograph of AhV VLPs and 2D class averages. Scale bar=100 nm. (**b**) The 2.4 Å resolution 3D reconstruction of AhV, colored according to quasi-conformer, with monomer A colored pink and monomer B colored grey. The capsid surface (left) and a central slice through the capsid (right) are shown. (**c**) The atomic model of AhV, showing the model for a single asymmetric unit from the bottom (left) and the side (right) with the monomers colored as in panel (b).

The overall capsid structure of the isometric AhV capsid is similar to the available structures of the picobirnavirus lineage of dsRNA viruses [40], including all PVs characterized to date. The capsid is composed of 120 coat proteins arranged in *T*=1 symmetry, where each asymmetric unit comprises a homodimer of the AhV CP (Fig. 2). Structure refinement with icosahedral symmetry imposed yielded a density map with a global resolution of 2.4 Å (Figs 2 and S2). The final atomic model was refined in the presence of surrounding chains to satisfy inter-chain interactions and revealed subtle differences between the individual chains of the asymmetric unit. The model for chain A includes 566 residues (71-268, 275-590 and 601-652) and chain B includes 562 residues (69-267, 274-537, 542-589 and 601-651). The N-termini of both chains consist of a long disordered region that has been seen in other PVs. The structure resolves a portion of the N-termini that could not be identified by mass spectroscopy, providing some indication, along with the apparent full-length protein on reducing SDS-PAGE, that the N-terminus is present. For both chains, short loops that are missing in the model occur on the surface of the capsid and are presumably too flexible to reliably model. Superposition of the individual chains yields an root mean square deviation (RMSD) of 0.4 Å (Fig. S3). Variations in backbone can be seen for short loops near the fivefold and threefold symmetry axes of the capsid, as would be anticipated for quasi-symmetric monomers.

**Fig. 3.**
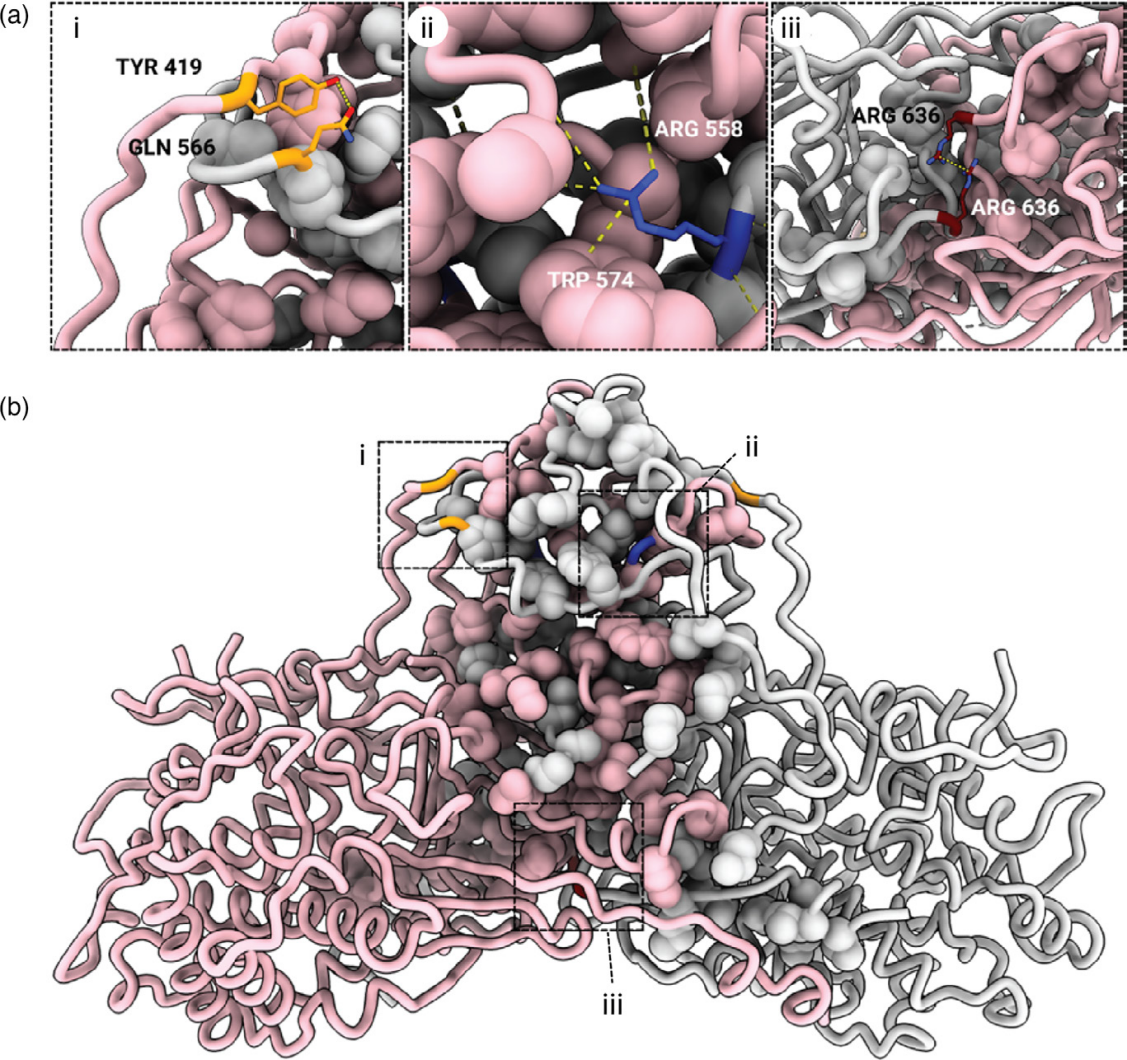
AhV CP interface. (**a**) Detail expansions corresponding to dashed boxes in B showing (i) the GLN566 to TYR419 hydrogen bond anchoring an extended invading loop from the opposite monomer, (ii) the extensive contacts within the hydrophobic interface and (iii) the symmetrical ARG636 to ARG636 pairing. (**b**) Side view of the CP dimer with the hydrophobic residues of the dimer interface shown as space-filling models. Monomer A is colored pink and monomer B is colored grey.

The AhV CP forms a *T*=1 icosahedron of 60 dimeric protomers comprised of two distinct domains: the shell (S) and protrusion (P) domains. The S domain (res. 71–409) adopts a predominantly *α*-helical fold with seven *α*-helices (*α*1-*α*7) arranged around a central, extended 24-residue helix (*α*3, res. 125–148). This arrangement creates a roughly rhomboidal shape that is characteristic of PV shell domains (Figs 2c and S4). The N-terminal helix *α*2 (res. 98–111) contains ARG111, which forms a stabilizing salt bridge with GLU75, anchoring the N-terminal region to the underside of the shell domain of the opposing monomer. Domain swapping by the CP N-terminus is a feature that appears to be common among the PVs. In betapartitiviruses, the N-terminus also contributes to the dimerization interface, with a largely hydrophobic contact patch (res. 71–85) involving ILE71, PHE72 and LEU78, complemented by the GLU75 to ARG111 salt bridge. Unlike the arch-forming P domain of the gammapartitiviruses [19,20] and the spike-like P domain of the deltapartitiviruses [17], the P domain of the betapartitiviruses takes the form of a butte (Fig. 1c), as previously described for capsid structures at lower resolution [14,41]. The high resolution of the AhV capsid reported here enables elaboration of the interactions that form the protrusion unique to the betapartitiviruses. The P domain (res. 410–652) constitutes the major dimerization interface, burying a substantial surface area of ~13,380 Å². The domain features a modified *β*-sheet structure and multiple interdigitated loops. The P domain achieves dimerization through an extensive hydrophobic interface that forms the core of the dimer interaction, anchored by hydrogen bonds, such as that between GLN566 and TYR419 [Fig. 3a(i)]. The central dimerization region contains multiple interaction hot spots, including ARG558 (24 contacts), LEU545 (22 contacts) and ASP564 (20 contacts), which create an extensive network of stabilizing interactions. The C-terminal interface region (res. 630–650) features unique interactions including a cation-π interaction between PHE633 and ARG636 and a symmetric ARG636 to ARG636 pairing between opposing monomers [Fig. 3a(ii)]. The CP dimer most likely represents the assembly unit for PVs [18]. However, the interactions between AhV CP monomers are more extensive and more varied than has previously been seen in the PVs. Further experimentation is required to determine whether the evolution of such a complex and tight binding interface is relevant to AhV biology beyond the structural role in the capsid. In the absence of a reverse genetic system for most persistent viruses like AhV, further investigation of the functions of PV proteins may be facilitated by recombinant expression approaches such as that described in this study.

### CP structures highlight considerable structural diversity among PVs

**Fig. 4.**
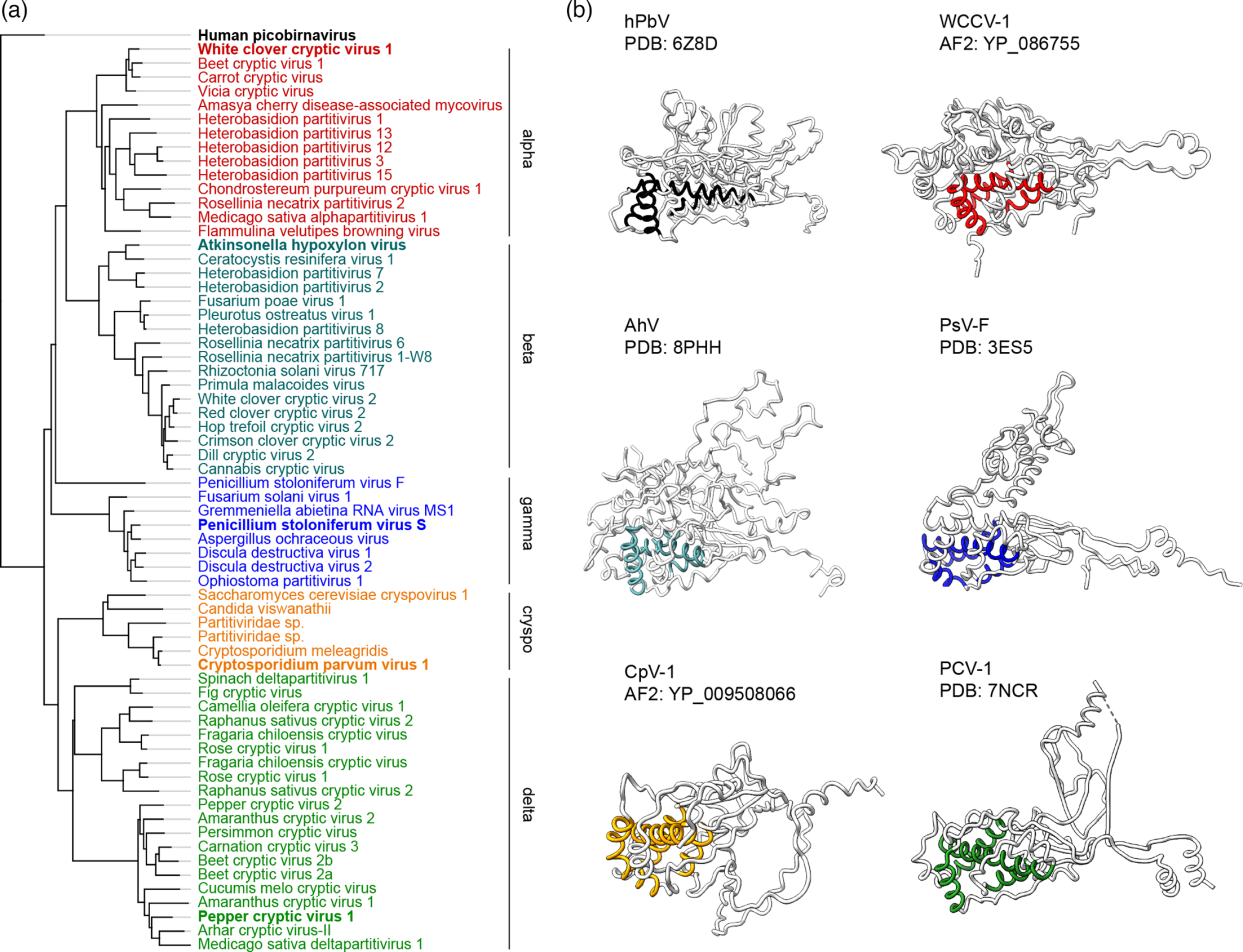
CP domain architecture in PVs. (**a**) Phylogram generated using IQ-TREE [31] and the Q.pfam+F+R4 evolution model [49] with the human picobrinavirus CP (YP_239360.1) used as an outgroup. The type member of each genus is shown in bold. A compressed tree is shown for convenience and the full tree with branch support values is presented in Fig. S5. (**b**) Molecular models of the CP monomers of representative members of each genus derived from empirically determined structures (indicated by PDB code) or predicted using AlphaFold2 (AF2; indicated by CP accession number). The color of the conserved helical core corresponds to the genus members shown in (a). hPbV is human picobirnavirus, WCCV-1 is white clover cryptic virus 1, AhV is *Atkinsonella hypoxylon virus*, PsV-F is *P. stoloniferum* virus F, CpV-1 is *C. parvum* virus 1 and PCV-1 is pepper cryptic virus 1.

Including the AhV CP structure reported here, four PV capsid structures have been resolved to high resolution: two gammapartitiviruses, and one each from the deltapartitiviruses and the betapartitiviruses. To investigate whether the remaining genera, specifically the alphapartitiviruses and cryspoviruses, have evolved yet different CP domain architectures, we used AlphaFold2 [42] to predict the structures for an additional 61 CP sequences. To generate a broader picture for each genus, we expanded the ICTV-defined family to include 6 cryspovirus CPs (an increase of 5 from the 1 formally recognized member; including 1 hypothetical protein from host genome sequencing) and 20 deltapartitiviruses (an increase of 15 from 5 formally recognized members), in addition to the formally recognized 17 alphapartitiviruses, 14 betapartitiviruses and 8 gammapartitiviruses (see Tables S1–S6). Using human picobirnavirus as an outgroup, phylogenetic analysis of the 65 PV CP sequences grouped the CPs into clades representing the expected genera based on the published RdRp phylogeny [1], with the exception of *Penicillium stoloniferum* virus S, which could not be reliably grouped with the gammapartitiviruses (Figs 4a and S5).

Structural alignment confirms the phylogenetic analysis of PV CP sequences with highly similar structures within genera and diverse CP architectures between them. For all CP predictions, the shell domain, particularly the core helices, was predicted with high (>90%) confidence (Figs S6–S11). The global confidence scores of the predicted structures varied considerably both within and between genera (Tables S1–S6), largely due to variable lengths of N-terminal, C-terminal or internal regions with lower local confidence scores (Figs S6–S11). The observed N-terminal disorder in PV CPs, internal disordered regions in the deltapartitiviruses [17] and domain swapping between CP dimers may contribute to lower confidence in the predicted structure of these regions of the monomers. Nevertheless, comparison of experimentally determined structures to their AlphaFold2 predictions shows very good alignment of the S domain with backbone RMSD values of 0.95 Å for the betapartitivirus AhV, 2.76 Å for the gammapartitivirus PsV-F and 1.03 Å for the deltapartitivirus PCV-1. Moreover, the orientation of major structural features for the betapartitiviruses, gammapartitiviruses and deltapartitiviruses was also correctly predicted (Figs S7, S8 and S10). It should be noted that neither the PCV-1 (7NCR) nor AhV (8PHH) structures were included in the training set of the AlphaFold version used. The predicted structures for alphapartitiviruses and cryspoviruses show that these genera also have differences in predicted CP domain architectures compared to the structurally characterized genera (Fig. 4b). The relatively short cryspovirus CP (Fig. S9) sequences possess the conserved core helices and an extended N-terminus but appear to consist of a minimal shell domain with no obvious protrusion. Alphapartitiviruses (Fig. S5) appear to have a mostly helical cap above the core helices and a long N-terminal loop that likely crosses the dimerization interface to interact with the other monomer of the presumed protomer, as is characteristic of the dimerization interactions within the family.

### Core helices are a highly conserved feature that accommodates structural diversity among PV CPs

**Fig. 5.**
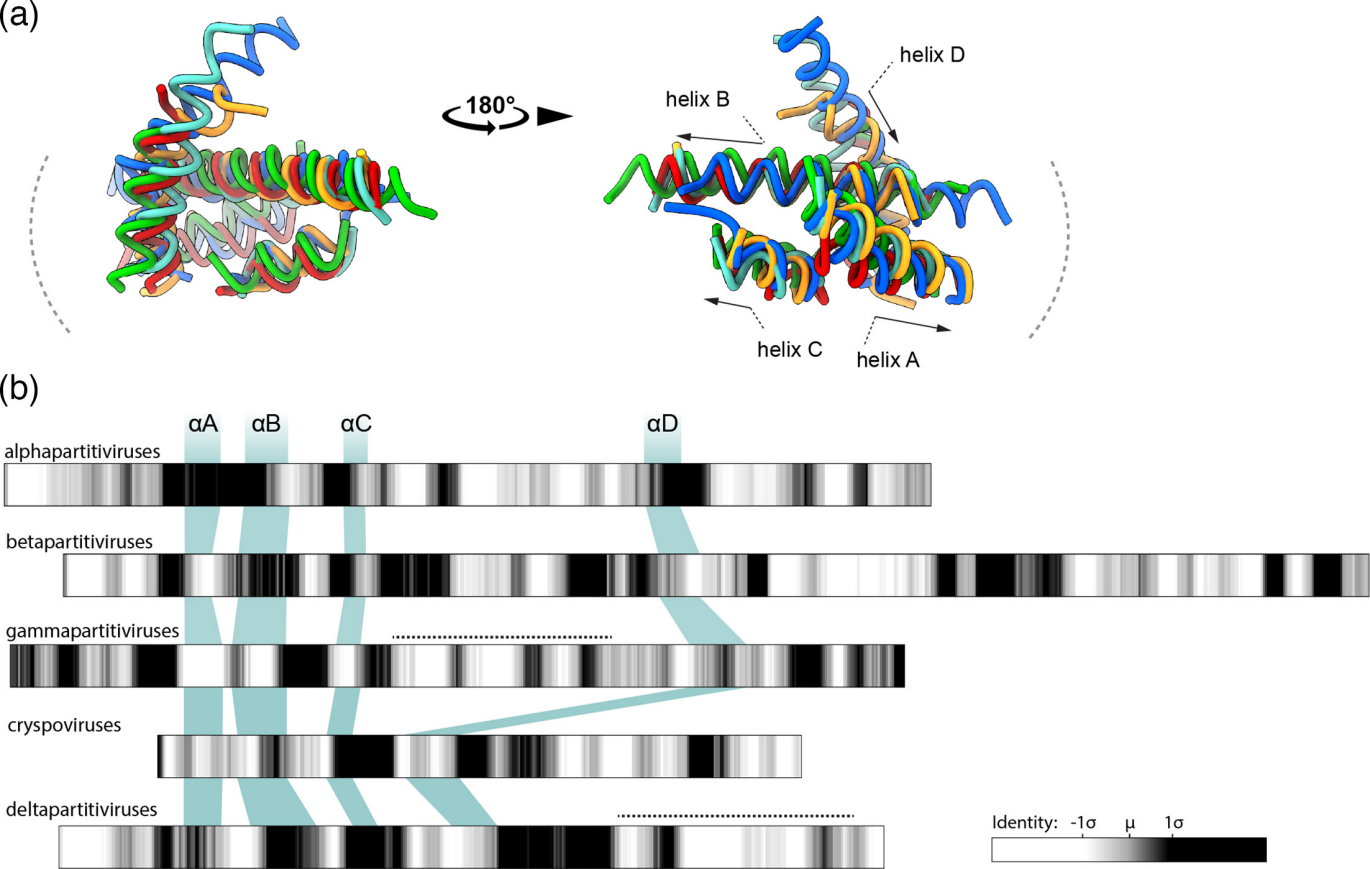
The conserved helical core of the shell domain in PVs. (**a**) Alignment of the core helices of representative CP structures from each recognized PV genus, annotated with the direction of the helix. The color of the conserved helical core corresponds to the individual genus members shown in Fig. 4(b): alphapartitivirus (AF2 – YP_086755) in red; betapartitivirus (PDB – 8PHH) in cyan; gammapartitivirus (PDB – 3ES5) in blue; cryspovirus (AF2 – YP_009508066) in yellow; and deltapartitivirus (PDB – 7NCR) in green. (**b**) Position of the core helices within the primary structure of each PV genus with the intra-genus sequence identity shown as a grey-scale heat map scaled to the mean identity calculated over a 10-residue sliding window. Dotted lines represent the protrusions in the gammapartitiviruses and deltapartitiviruses.

Although the overall architecture of the CP is strikingly different between PV genera, the shell domain of all the PV CPs analyzed contains a recognizable core of four *α*-helices (Fig. 4b). In the betapartitivirus, AhV, these helices correspond to *α*2, *α*3, *α*6 and *α*12 (Fig. S4). Previously identified in gammapartitiviruses and deltapartitiviruses [17], here, we show that it is present in all genera, in addition to the picobirnavirus CP, supporting the idea that it is a conserved element of the picobirnavirus CP structural lineage. Alignment of the core helices, hereafter termed *α*A-*α*D, shows that the 3D organization of the core, including the direction of the helices, is highly conserved (Fig. 5a). A central long helix (*α*B) forms the backbone of the core and it is crossed by an initial helix (*α*A) that runs away from the dimerization interface to the distal edge of the protomer and a smaller helix (*α*C) returning back towards the interface in the same direction as *α*B. A fourth helix (*α*D) runs perpendicular to the first three, running from the outside of the capsid to the inner surface. This organization also holds for human picobirnavirus (Fig. S12). These data indicate that the core helices may indeed comprise the minimal fold of the shell domain of the PVs [17], although it remains to be seen whether this holds for newly discovered PVs in insects and sister families in prokaryotes.

A subset of deltapartitiviruses has putative CPs that are shorter than the demarcation criteria of the genus. No virions or capsids have been observed for the so-called short form deltapartitiviruses. However, the predicted structures of putative CPs from the short form deltapartitiviruses possess the core helices and have the same organization and orientation as the other PVs (Fig. S11). This appears to support their role as putative CPs. Moreover, some short form deltapartitiviruses appear to be tripartite, with a second putative CP. In these cases, despite sharing as little as ~50% sequence identity, both CP-like sequences are predicted to have very similar structures (Table S6, Fig. S11).

Despite the structural conservation of the core helices, they do not correspond to regions of sequence conservation within genera (Fig. 5b). Moreover, their position in the primary sequence varies considerably between genera. In the deltapartitiviruses and cryspoviruses, the core helices are grouped together in the N-terminal half of the protein. In alphapartitiviruses, betapartitiviruses and gammapartitiviruses, *α*D is separated from the first three core helices by an insertion of ~150 amino acids (Fig. 5b). In both the experimentally derived betapartitivirus structure and predicted alphapartitivirus structure, the insertion consists of an *α*-helical ‘cap’ over the S domain. In the gammapartitiviruses, the insertion forms the mostly helical P domain that forms an arch over the CP dimer. This is in stark contrast to the deltapartitivirus P domain at the dimerization interface and entirely C-terminal to the core helices (Fig. 5b). In AhV and other betapartitiviruses, the protrusion forming the dimerization interface is also C-terminal to the final core helix αD, albeit discontinuous with elements of the S domain cap. As has been noted for other dsRNA viruses [43], it is tempting to speculate that the core helices provide an evolutionarily conserved fold that allows for the insertion of functional sequences and domains.

Viruses with persistent lifestyles and exclusively vertical transmission, such as PVs, are predicted to evolve towards maximizing the reproduction of their hosts [44]. Evidence of mutualism in plant PVs [11,12] and ecological effects on fungal hosts [13,14, 16] indicate host adaptation, but the viral determinants are not known. It has been shown that PV RdRps are functional across host kingdoms [45,46], implying that replication and transcription may not restrict host range. The CPs of dsRNA viruses are known to acquire auxiliary functions that mediate host interactions, such as cap-snatching [47] and possibly other enzymatic activities [43]. In addition, the CPs of many RNA viruses are known to regulate transcription and translation of their hosts. Therefore, although ascribing adaptive traits that enable transfer to new hosts within the RdRp cannot be ruled out, the CP of PVs may play an important role.

## Conclusions

The high-resolution structure of the AhV capsid reported here confirms that the betapartitiviruses share a consistent CP and capsid organization with other PVs. It reveals an extensive hydrophobic dimerization interface that consists of multiple penetrating loops from opposite dimers, anchored by hydrogen bonds. It appears to be a highly evolved interface, including a symmetrical interaction between dimers, that reinforces the essential role of the dimer in capsid assembly. Analysis of the CPs, including structural prediction, across the recognized genera of the PVs shows that there is a high level of intra-genus structural relatedness and divergent architectures between genera. The family is characterized by a conserved shell constructed around a helical core that appears to be characteristic of the CP fold in the picobirnavirus lineage. Our results show that the shell domain, particularly the core helices, represents a structural template that the *Partitiviridae* have used to elaborate a variety of protrusions that define the genera. A similar diversification of CP architectures on a conserved shell domain was recently described within a proposed family of dsRNA marine bacteriophages, the *Paraxenoviridae* [48]. Therefore, it seems to be a general strategy of viruses in the picobirnavirus CP lineage. In the *Partitiviridae*, unlike the genera for which high-resolution structures previously existed, the *Deltapartitivirus* and *Gammapartitivirus*, the genus *Betapartitivirus* includes members with both plant and fungal hosts. It is, therefore, interesting for the study of viruses that may have undergone horizontal virus transfer, as has been suggested in PVs by phylogenetic analysis. The establishment of infections in heterologous or model hosts could help shed light on the virus–host interactions that restrict or enable host range expansion. A complementary approach is structural virology, and here, we have established a reference structure for this genus.

## Supplementary material

10.1099/jgv.0.002209 Uncited Supplementary Material 1.

## Abbreviations

AhV: *Atkinsonella hypoxylon* virus
CP: capsid protein
PV: partitivirus
RdRp: RNA-dependent RNA polymerase

## References

[1] Vainio EJ, Chiba S, Ghabrial SA, Maiss E, Roossinck M (2018). ICTV Virus Taxonomy Profile: partitiviridae. J Gen Virol.

[2] Taggart NT, Crabtree AM, Creagh JW, Bizarria R, Li S (2023). Novel viruses of the family partitiviridae discovered in *Saccharomyces cerevisiae*. PLOS Pathog.

[3] Nerva L, Silvestri A, Ciuffo M, Palmano S, Varese GC (2017). Transmission of *Penicillium aurantiogriseum* partiti-like virus 1 to a new fungal host (*Cryphonectria parasitica*) confers higher resistance to salinity and reveals adaptive genomic changes. Environ Microbiol.

[4] Gilbert KB, Holcomb EE, Allscheid RL, Carrington JC (2019). Hiding in plain sight: New virus genomes discovered via a systematic analysis of fungal public transcriptomes. PLOS One.

[5] Jiang Y, Wang J, Yang B, Wang Q, Zhou J (2019). Molecular characterization of a debilitation-associated partitivirus infecting the pathogenic fungus *Aspergillus flavus*. Front Microbiol.

[6] Neri U, Wolf YI, Roux S, Camargo AP, Lee B (2022). Expansion of the global RNA virome reveals diverse clades of bacteriophages. Cell.

[7] Urayama S, Fukudome A, Hirai M, Okumura T, Nishimura Y (2024). Double-stranded RNA sequencing reveals distinct riboviruses associated with thermoacidophilic bacteria from hot springs in Japan. Nat Microbiol.

[8] Le Lay C, Stott MB, Shi M, Sadiq S, Holmes EC (2023). A metatranscriptomic analysis of geothermal hot springs reveals diverse RNA viruses including the phylum lenarviricota. Virology.

[9] Turner D, Adriaenssens EM, Amann RI, Bardy P, Bartlau N (2025). Summary of taxonomy changes ratified by the International Committee on Taxonomy of Viruses (ICTV) from the bacterial viruses subcommittee, 2025. J Gen Virol.

[10] Roossinck MJ (2012). Plant virus metagenomics: biodiversity and ecology. Annu Rev Genet.

[11] Safari M, Ferrari MJ, Roossinck MJ (2019). Manipulation of aphid behavior by a persistent plant virus. J Virol.

[12] Nakatsukasa-Akune M, Yamashita K, Shimoda Y, Uchiumi T, Abe M (2005). Suppression of root nodule formation by artificial expression of the TrEnodDR1 (coat protein of White clover cryptic virus 1) gene in *Lotus japonicus*. Mol Plant Microbe Interact.

[13] Guo J, Zhang P, Wu N, Liu W, Liu Y (2024). Transfection of entomopathogenic *Metarhizium* species with a mycovirus confers hypervirulence against two lepidopteran pests. Proc Natl Acad Sci USA.

[14] Xiao X, Cheng J, Tang J, Fu Y, Jiang D (2014). A novel partitivirus that confers hypovirulence on plant pathogenic fungi. J Virol.

[15] Zheng L, Zhang M, Chen Q, Zhu M, Zhou E (2014). A novel mycovirus closely related to viruses in the genus *Alphapartitivirus* confers hypovirulence in the phytopathogenic fungus *Rhizoctonia solani*. Virology.

[16] Hyder R, Pennanen T, Hamberg L, Vainio EJ, Piri T (2013). Two viruses of *Heterobasidion* confer beneficial, cryptic or detrimental effects to their hosts in different situations. Fungal Ecology.

[17] Byrne M, Kashyap A, Esquirol L, Ranson N, Sainsbury F (2021). The structure of a plant-specific partitivirus capsid reveals a unique coat protein domain architecture with an intrinsically disordered protrusion. *Commun Biol*.

[18] Ochoa WF, Havens WM, Sinkovits RS, Nibert ML, Ghabrial SA (2008). Partitivirus structure reveals a 120-subunit, helix-rich capsid with distinctive surface arches formed by quasisymmetric coat-protein dimers. Structure.

[19] Pan J, Dong L, Lin L, Ochoa WF, Sinkovits RS (2009). Atomic structure reveals the unique capsid organization of a dsRNA virus. Proc Natl Acad Sci USA.

[20] Tang J, Pan J, Havens WM, Ochoa WF, Guu TSY (2010). Backbone trace of partitivirus capsid protein from electron cryomicroscopy and homology modeling. Biophys J.

[21] Oh C-S, Hillman BI (1995). Genome organization of a partitivirus from the filamentous ascomycete *Atkinsonella hypoxylon*. J Gen Virol.

[22] Sainsbury F, Thuenemann EC, Lomonossoff GP (2009). PEAQ: versatile expression vectors for easy and quick transient expression of heterologous proteins in plants. Plant Biotechnol J.

[23] Kimanius D, Dong L, Sharov G, Nakane T, Scheres SHW (2021). New tools for automated cryo-EM single-particle analysis in RELION-4.0. Biochem J.

[24] Zheng SQ, Palovcak E, Armache J-P, Verba KA, Cheng Y (2017). MotionCor2: anisotropic correction of beam-induced motion for improved cryo-electron microscopy. Nat Methods.

[25] Zhang K (2016). Gctf: Real-time CTF determination and correction. J Struct Biol.

[26] Jamali K, Käll L, Zhang R, Brown A, Kimanius D (2024). Automated model building and protein identification in cryo-EM maps. Nature.

[27] Emsley P, Cowtan K (2004). Coot: model-building tools for molecular graphics. Acta Crystallogr D Biol Crystallogr.

[28] Adams PD, Afonine PV, Bunkóczi G, Chen VB, Davis IW (2010). PHENIX: a comprehensive Python-based system for macromolecular structure solution. Acta Crystallogr D Biol Crystallogr.

[29] Davis IW, Leaver-Fay A, Chen VB, Block JN, Kapral GJ (2007). MolProbity: all-atom contacts and structure validation for proteins and nucleic acids. Nucleic Acids Res.

[30] Pettersen EF, Goddard TD, Huang CC, Meng EC, Couch GS (2021). UCSF ChimeraX: structure visualization for researchers, educators, and developers. Protein Sci.

[31] Minh BQ, Schmidt HA, Chernomor O, Schrempf D, Woodhams MD (2020). IQ-TREE 2: new models and efficient methods for phylogenetic inference in the genomic era. Mol Biol Evol.

[32] Kalyaanamoorthy S, Minh BQ, Wong TKF, von Haeseler A, Jermiin LS (2017). ModelFinder: fast model selection for accurate phylogenetic estimates. Nat Methods.

[33] The Galaxy C (2024). The Galaxy platform for accessible, reproducible, and collaborative data analyses: 2024 update. Nucleic Acids Res.

[34] Hoang DT, Chernomor O, von Haeseler A, Minh BQ, Vinh LS (2018). UFBoot2: improving the ultrafast bootstrap approximation. Mol Biol Evol.

[35] Byrne MJ, Steele JFC, Hesketh EL, Walden M, Thompson RF (2019). Combining transient expression and Cryo-EM to obtain high-resolution structures of luteovirid particles. Structure.

[36] Hesketh EL, Saunders K, Fisher C, Potze J, Stanley J (2018). The 3.3 Å structure of a plant geminivirus using cryo-EM. Nat Commun.

[37] Castells-Graells R, Ribeiro JRS, Domitrovic T, Hesketh EL, Scarff CA (2021). Plant-expressed virus-like particles reveal the intricate maturation process of a eukaryotic virus. Commun Biol.

[38] Marsian J, Hurdiss DL, Ranson NA, Ritala A, Paley R (2019). Plant-made nervous necrosis virus-like particles protect fish against disease. Front Plant Sci.

[39] Marsian J, Fox H, Bahar MW, Kotecha A, Fry EE (2017). Plant-made polio type 3 stabilized VLPs-a candidate synthetic polio vaccine. Nat Commun.

[40] Abrescia NGA, Bamford DH, Grimes JM, Stuart DI (2012). Structure unifies the viral universe. Annu Rev Biochem.

[41] Tang J, Ochoa WF, Li H, Havens WM, Nibert ML (2010). Structure of Fusarium poae virus 1 shows conserved and variable elements of partitivirus capsids and evolutionary relationships to picobirnavirus. J Struct Biol.

[42] Jumper J, Evans R, Pritzel A, Green T, Figurnov M (2021). Highly accurate protein structure prediction with AlphaFold. Nature.

[43] Mata CP, Luque D, Gómez-Blanco J, Rodríguez JM, González JM (2017). Acquisition of functions on the outer capsid surface during evolution of double-stranded RNA fungal viruses. PLOS Pathog.

[44] García-Ordóñez L, Pagán I (2024). Vertical and horizontal transmission of plant viruses: two extremes of a continuum?. *Npj Viruses*.

[45] Nerva L, Varese GC, Falk BW, Turina M (2017). Mycoviruses of an endophytic fungus can replicate in plant cells: evolutionary implications. Sci Rep.

[46] Telengech P, Hyodo K, Ichikawa H, Kuwata R, Kondo H (2024). Replication of single viruses across the kingdoms, Fungi, Plantae, and Animalia. Proc Natl Acad Sci USA.

[47] Fujimura T, Esteban R (2011). Cap-snatching mechanism in yeast L-A double-stranded RNA virus. Proc Natl Acad Sci USA.

[48] Yoshida M, Medvedeva S, Fukudome A, Wolf YI, Urayama S-I (2025). “Paraxenoviridae”, a putative family of globally distributed marine bacteriophages with double-stranded RNA genomes. ISME J.

[49] Minh BQ, Dang CC, Vinh LS, Lanfear R (2021). QMaker: fast and accurate method to estimate empirical models of protein evolution. Syst Biol.

