# Supplementary material for: Atkinsonella hypoxylon virus capsid structure highlights the diversity of capsid proteins among the Partitiviridae
Source: J Gen Virol. 2026 Jan 16;107(1):002209. doi: 10.1099/jgv.0.002209 (PMC12811077; doi:10.1099/jgv.0.002209)
Supplement: Uncited Supplementary Material 1. [file jgv-107-02209-s001.pdf]

SUPPLEMENTARY DATA

Atkinsonella hypoxylon virus capsid structure highlights the diversity of capsid proteins among the *Partitiviridae*

Micol Venturi[1]*, Matthew Calthorpe-Byrne[2]*, Beate Aftret[1], Donna McNeale[1], Bernd Rehm[1], Frank Sainsbury[1,3]†

[1]Centre for Cell Factories and Biopolymers, Griffith Institute for Drug Discovery, Griffith University, Nathan, QLD 4111, Australia
[2]electron Bio-Imaging Centre, Diamond Light Source Ltd, Didcot, Oxfordshire OX11 0DE, United Kingdom
[3]Australian Research Council Centre of Excellence in Synthetic Biology

* Equal contribution
† Correspondence: f.sainsbury@griffith.edu.au

Contents:

Figure S1 – Sequence coverage by peptide mass fingerprinting

Figure S2 – Local resolution and Fourier shell correlation

Figure S3 – Superposition of chain A and chain B monomers

Figure S4 – Capsid protein secondary structure composition

Figure S5 – Phylogram of the Partitiviruses based on CP sequence

Figure S6 – *Alphapartitivirus* structural alignment

Table S1 – *Alphapartitivirus* CP sequences included in this study

Figure S7 – *Betapartitivirus* structural alignment

Table S2 – *Betapartitivirus* CP sequences included in this study

Figure S8 – *Gammapartitivirus* structural alignment

Table S3 – *Gammapartitivirus* CP sequences included in this study

Figure S9 – *Cryspovirus* structural alignment

Table S4 – *Cryspovirus* CP sequences included in this study

Figure S10 –*Deltapartitivirus* (long form CP) structural alignment

Table S5 – *Deltapartitivirus* (long form CP) sequences included in this study

Figure S11 – Structural analysis of short form CP deltapartitiviruses

Table S6 – Short form *Deltapartitivirus* CP sequences included in this study

Figure S12 – Conservation of core helices including human picobirnavirus CP

```
MSSNDSAQTRNLQEERFNERTSTPTVVTAVLPDTNGPTTNSTSGSVGPPHPTPNVPVPTQ
SSSDPPSASGIFAKEIDLPRNVIQHSGNKFILDVVPDSRFPTFAITEFVQRSFSNFTFEQ
YSYVSPASLVGYLVYMIHAFVFLVDAFERSPMSAYASEIDASHAYLRIIDAFSDAYIPDF
LFEILDTYLSHRLDIRSKLEMNVSYGSVLYKYDAPRIVAPSIFLLAHNQLISQSRESTAY
EKWLDSIVIHYSRAVIRVGNLVGGLYQSSHGSTTTHFTYRNWFARSLSRLADSATHRTHL
RRPMISEFDYNIPSVNNNTYNPYVHLLMLEPNNRNITLDFIRSLSSFCSTELKATRTLRD
HISRRSAAISRCVIKGPEAPTWHSSPLDDLKEKSKQGNFSQFCEVAKFGLPRKENSESYT
FKFPKDASTIDTAFYLIQENGRSSVLDPTTADEELHTEGMNLLFDPYDDESSAHYATVLS
GKLIQNSNIDGETLLLPDPTTGLARTNSRYLQGSVLIRNVLPEFDQHEIRLFPRYPQISR
LSASLTLLFNMRQVWIPRFKQKVDEQPKLSNFSWNEGCDGTVPSLNVVTAESSTNGPAAE
QQVILWSSYRHVSNSDRPTVDTVYYYSTLELLFGTRSSMMQTYNLHQLLSLH
```

Figure S1. Sequence coverage by peptide mass fingerprinting.

Coverage is indicated in red for the peptides identified following in-gel trypsin digest of the AhV CP.

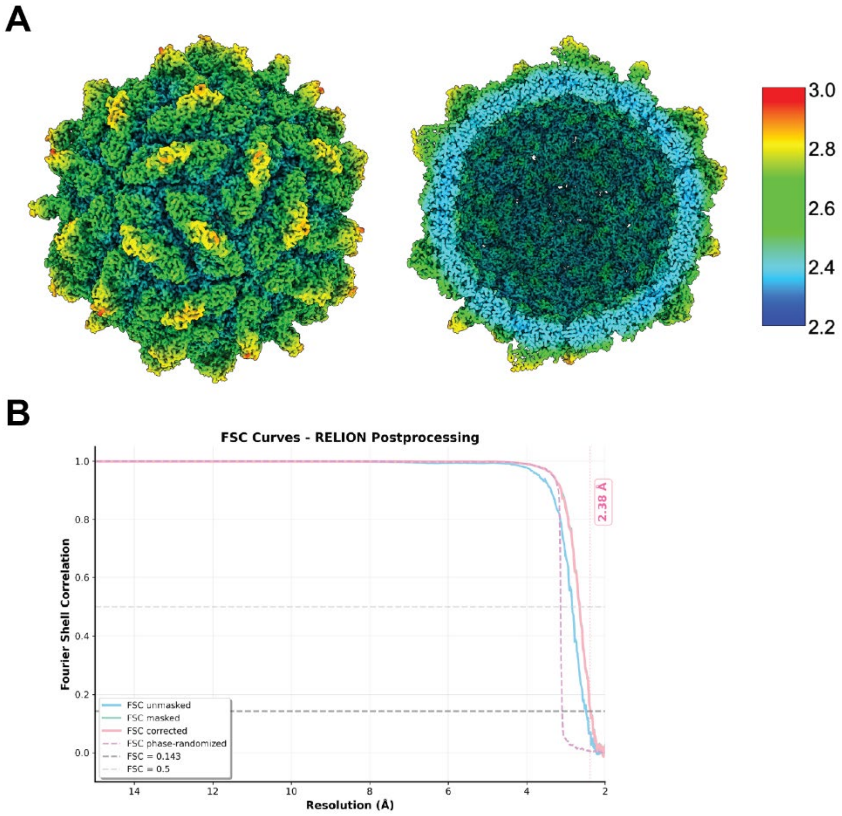

Figure S2. Local resolution and Fourier shell correlation.

(A) Local resolution filtered map of AhV VLP coloured according to resolution, showing capsid surface (left), and a central slice (right). **(B)** Fourier shell correlation – the resolution that corresponds to an FSC coefficient of 0.143 is 2.4 Å.

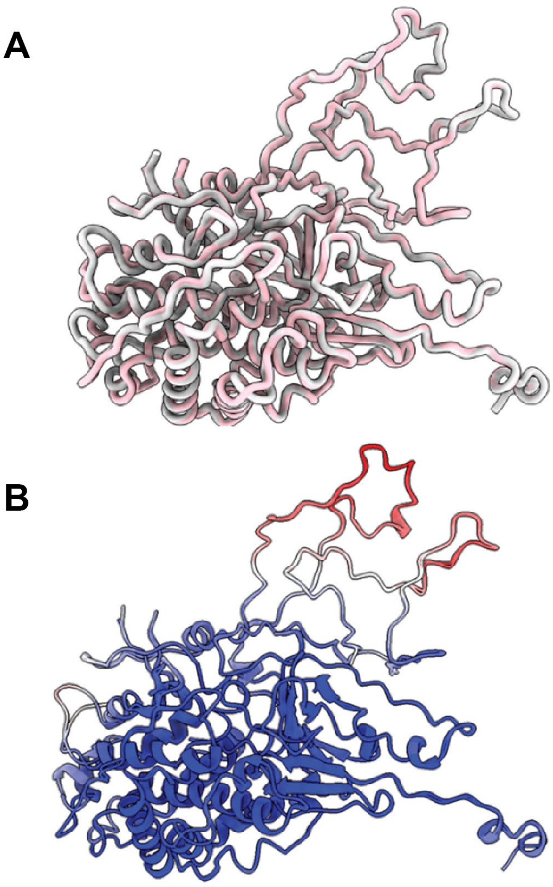

Figure S3. Superposition of chain A and chain B monomers.

Superposition of chain A and chain B from a single asymmetric unit of the AhV capsid. **(A)** Superposed monomers, shown as backbone ribbons for clarity. Coloured according to chain with chain A coloured pink, and chain b coloured grey. **(B)** Superposed AhV monomers, coloured according to B factor, from low (blue) to high (red).

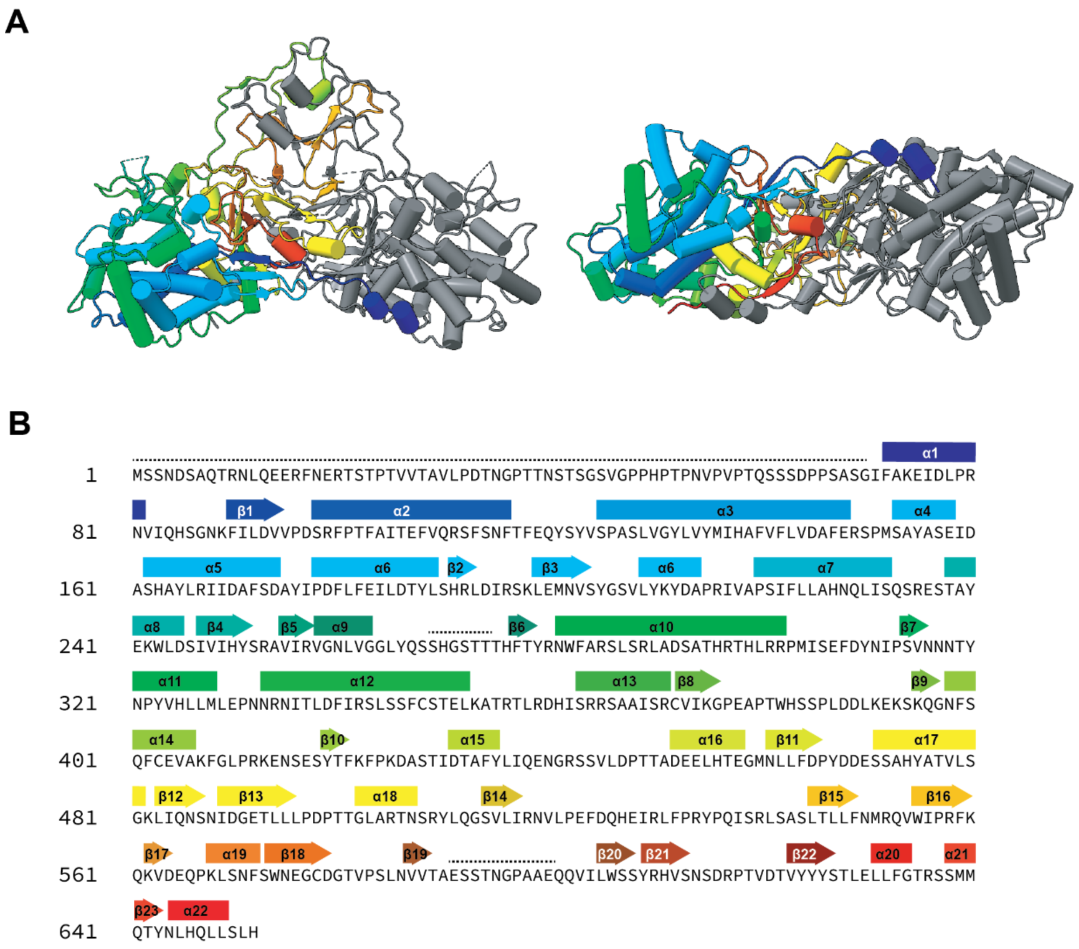

Figure S4. Capsid protein secondary structure composition.

(A) AhV CP shown in cartoon form viewed from the side (left) and the bottom (right) with monomer A coloured from N (blue) to C terminus (red). Monomer B is coloured grey for clarity. **(B)** AhV CP sequence labelled with secondary structure elements from chain A. Regions of disorder, that are not present in the final 3D reconstruction are denoted by a dashed line.

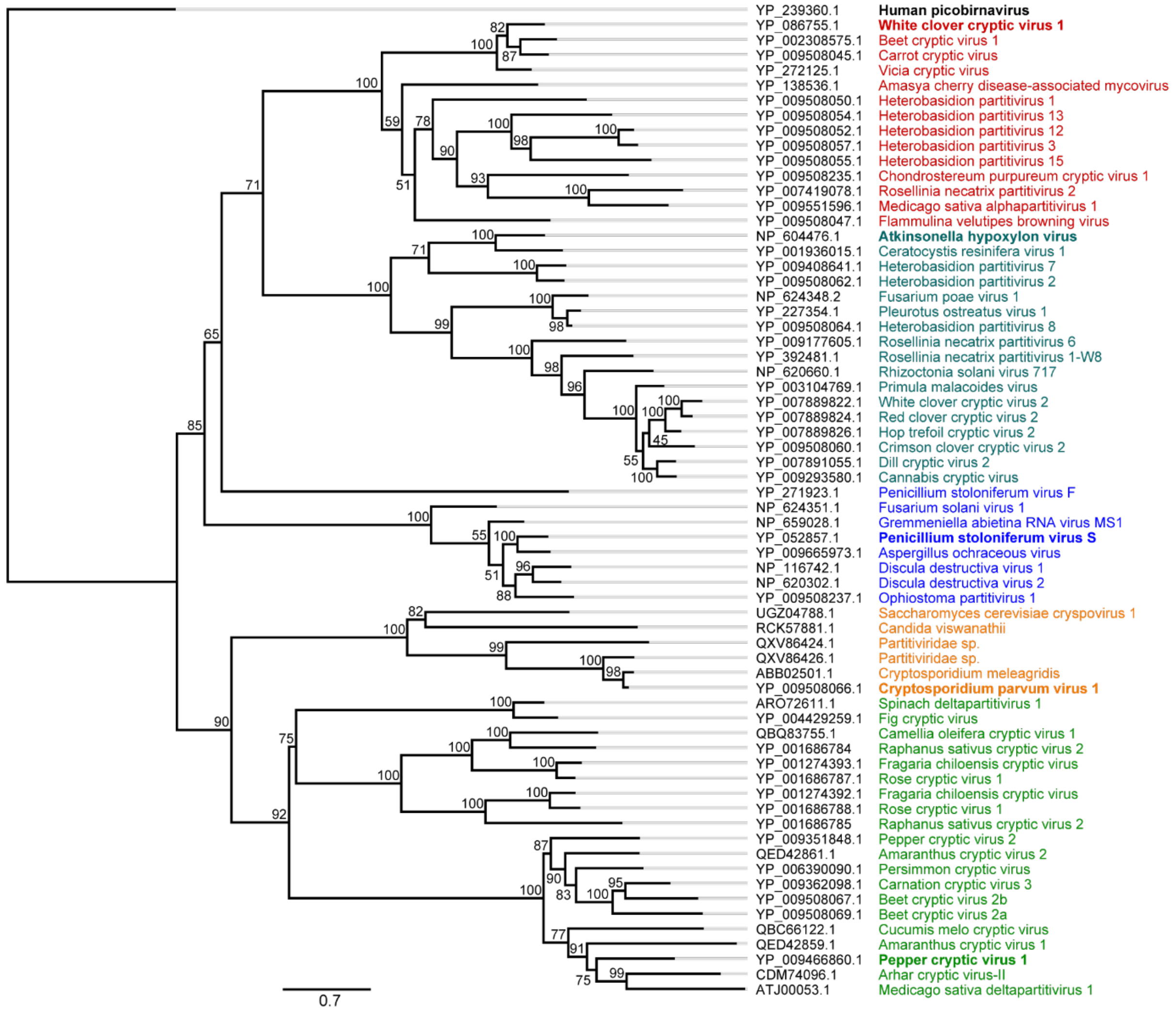

Figure S5. Phylogram of the Partitiviruses based on CP sequence.

The maximum likelihood tree was constructed IQ-Tree (1) and the Q.pfam+F+R4 evolution model (2). The initial alignment was created using the MAFFT algorithm L-INS-I and columns where at least 40% of the sequences had gaps were masked. Branch support is indicated at internal nodes and was determined using UFBoot2 (3) with values given as a percentage of 1000 replicates. The typical member of each genus is shown in bold.

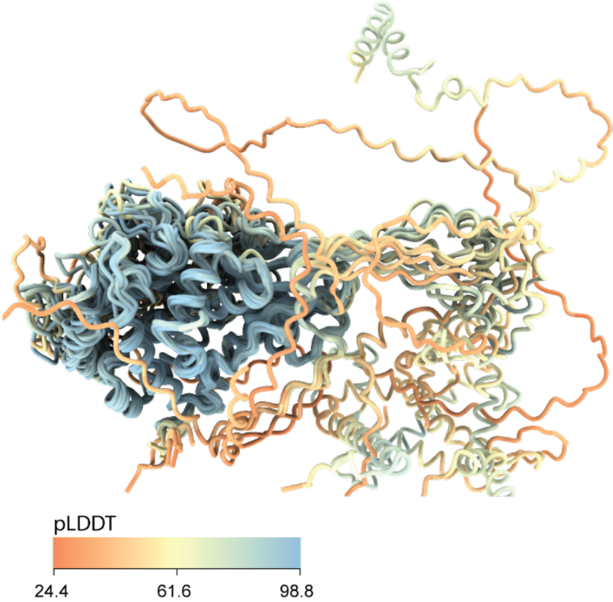

Figure S6. ***Alphapartitivirus*** **structural alignment.**

The predicted CP sequences of alphapartitiviruses were aligned using the matchmaker command in ChimeraX version 1.6 using white clover cryptic virus 1 as the reference. The structures include those predicted by alphafold2 (Table S1) and are coloured by the predicted local distance difference test (pLDDT) scores.

Table S1. ***Alphapartitivirus*** **CP sequences used in this study.**

Accession	Virus name	Length (aa)	pLDDT[a]
YP_009508047.1	Flammulina velutipes browning virus	463	n/a
YP_009508235.1	Chondrostereum purpureum cryptic virus 1	480	77.52
YP_007419078.1	Rosellinia necatrix partitivirus 2	483	81.91
YP_086755.1	White clover cryptic virus 1	487	85.27
YP_272125.1	Vicia cryptic virus	487	84.20
YP_002308575.1	Beet cryptic virus 1	489	84.65
YP_009508045.1	Carrot cryptic virus	490	86.05
YP_009551596.1	Medicago sativa alphapartitivirus 1	491	89.33
YP_138536.1	Amasya cherry disease-associated mycovirus	504	81.57
YP_009508054.1	Heterobasidion partitivirus 13	509	79.89
YP_009508050.1	Heterobasidion partitivirus 1	510	73.76
YP_009508055.1	Heterobasidion partitivirus 15	512	81.52
YP_009508052.1	Heterobasidion partitivirus 12	520	81.82
YP_009508057.1	Heterobasidion partitivirus 3	521	80.88

a - global score for model 1 in all cases, except ‘n/a’ where a result was not returned

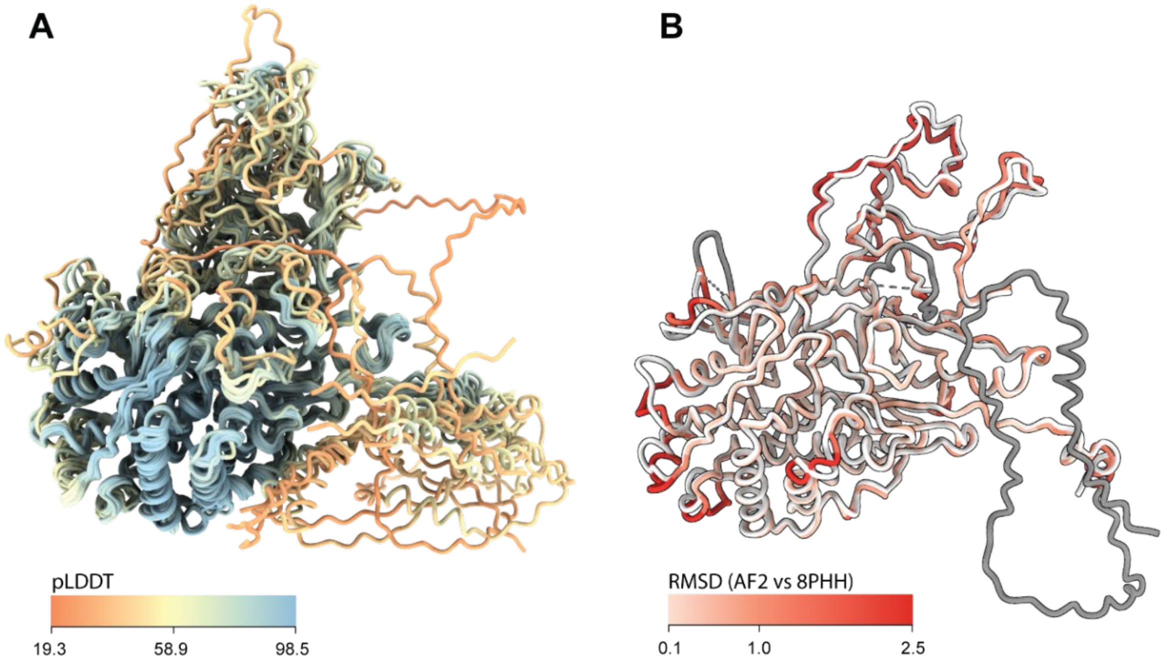

Figure S7. ***Betapartitivirus*** **structural alignment.**

(A) The predicted CP sequences of betapartitiviruses were aligned using the matchmaker command in ChimeraX version 1.6 using atkinsonella hypolxylon virus (AhV) as the reference. The structures include those predicted by alphafold2 (Table S2 below) and are coloured by the predicted local distance difference test (pLDDT) scores. **(B)** Alignment of the alphafold2 prediction and experimentally determined AhV CP structure (PDB 8PHH), coloured by root mean square deviation.

Table S2. ***Betapartitivirus*** **CP sequences used in this study.**

Accession	Virus name	Length (aa)	pLDDT[a]
YP_227354.1	Pleurotus ostreatus virus 1	636	80.97
NP_624348.2	Fusarium poae virus 1	637	83.19
YP_009508064.1	Heterobasidion partitivirus 8	638	81.47
NP_604476.1	Atkinsonella hypoxylon virus	652	80.54
YP_009408641.1	Heterobasidion partitivirus 7	654	77.69
YP_009508062.1	Heterobasidion partitivirus 2	659	78.37
YP_001936015.1	Ceratocystis resinifera virus 1	661	73.38
YP_009293580.1	Cannabis cryptic virus	672	86.86
YP_003104769.1	Primula malacoides virus	673	86.49
YP_007889822.1	White clover cryptic virus 2	673	86.13
YP_007889824.1	Red clover cryptic virus 2	673	84.90
YP_007889826.1	Hop trefoil cryptic virus 2	673	84.99
YP_007891055.1	Dill cryptic virus 2	673	n/a
YP_009508060.1	Crimson clover cryptic virus 2	674	86.56
NP_620660.1	Rhizoctonia solani virus 717	683	77.21
YP_392481.1	Rosellinia necatrix partitivirus 1-W8	686	74.46
YP_009177605.1	Rosellinia necatrix partitivirus 6	729	71.08

a - global score for model 1 in all cases, except 'n/a' where a result was not returned

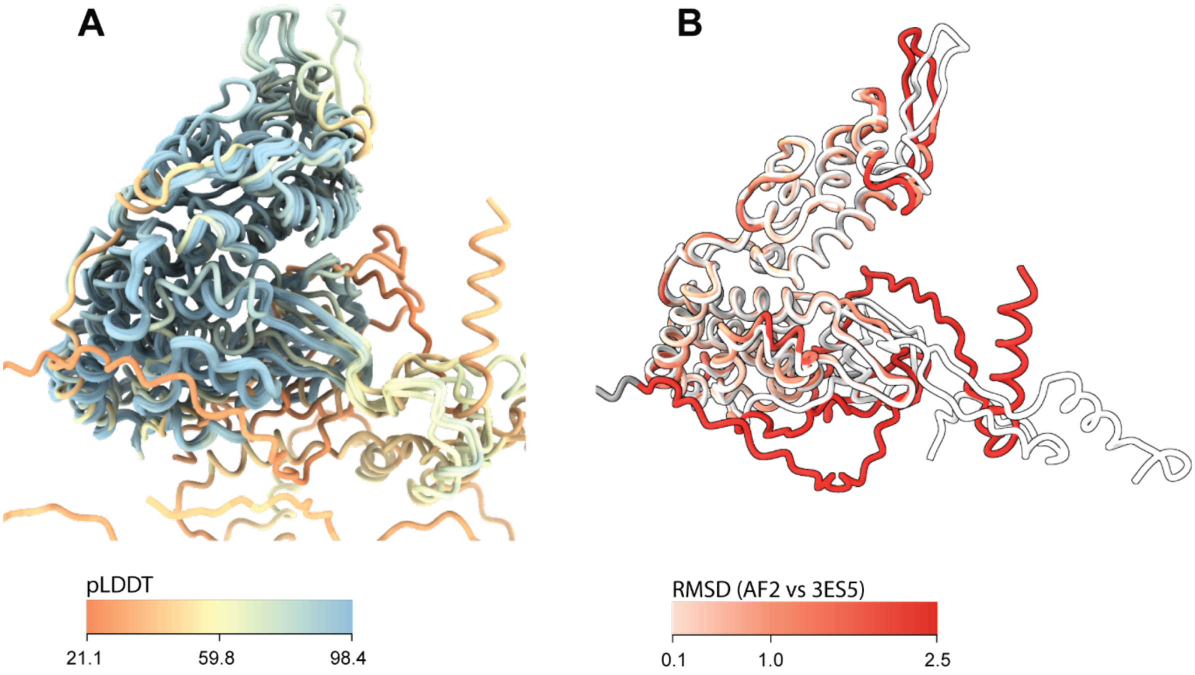

Figure S8. *Gammapartitivirus* structural alignment.

(A) The predicted CP sequences of gammapartitiviruses were aligned using the matchmaker command in ChimeraX version 1.6 using penicillium stoloniferum virus F (PsV-F) as the reference. The structures include those predicted by alphafold2 (Table S3 below) and are coloured by the predicted local distance difference test (pLDDT) scores. **(B)** Alignment of the alphafold2 prediction and experimentally determined PsV-F CP structure (PDB 3ES5), coloured by root mean square deviation.

Table S3. *Gammapartitivirus* CP sequences used in this study.

Accession	Virus name	Length (aa)	pLDDT[a]
NP_624351.1	Fusarium solani virus 1	413	61.31
YP_271923.1	Penicillium stoloniferum virus F	420	64.25
YP_009508237.1	Ophiostoma partitivirus 1	430	86.53
YP_009665973.1	Aspergillus ochraceous virus	433	84.73
YP_052857.1	Penicillium stoloniferum virus S	434	85.14
NP_116742.1	Discula destructiva virus 1	436	84.35
NP_620302.1	Discula destructiva virus 2	442	85.20
NP_659028.1	Gremmeniella abietina RNA virus MS1	443	84.66

a - global score for model 1 in all cases

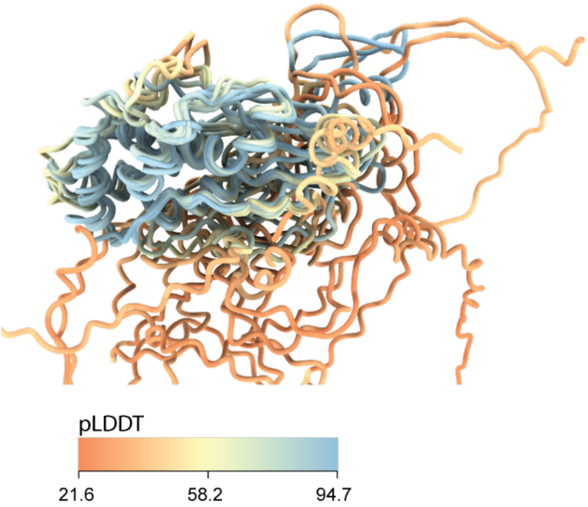

Figure S9. *Cryspovirus* structural alignment.

The predicted CP sequences of cryspoviruses were aligned using the matchmaker command in ChimeraX version 1.6 using cryptosporidium parvum virus 1 as the reference. The structures include those predicted by alphafold2 (Table S4 below) and are coloured by the predicted local distance difference test (pLDDT) scores.

Table S4. *Cryspovirus* CP sequences used in this study.

Accession	Virus name	Length (aa)	pLDDT[a]
RCK57881.1	Candida viswanathi virus[b,c]	307	78.83
UGZ04788.1	Saccharomyces cerevisiae cryspovirus 1[b]	310	70.06
ABB02501.1	Cryptosporidium meleagridis virus[b]	319	51.86
YP_009508066.1	Cryptosporidium parvum virus 1	319	54.84
QXV86424.1	Partitiviridae sp.[b]	358	56.89
QXV86426.1	Partitiviridae sp.[b]	366	50.67

a – global score for model 1 in all cases

b - not formally assigned to cryspoviruses

c - derived from genome sequence of *Candida viswanathii*

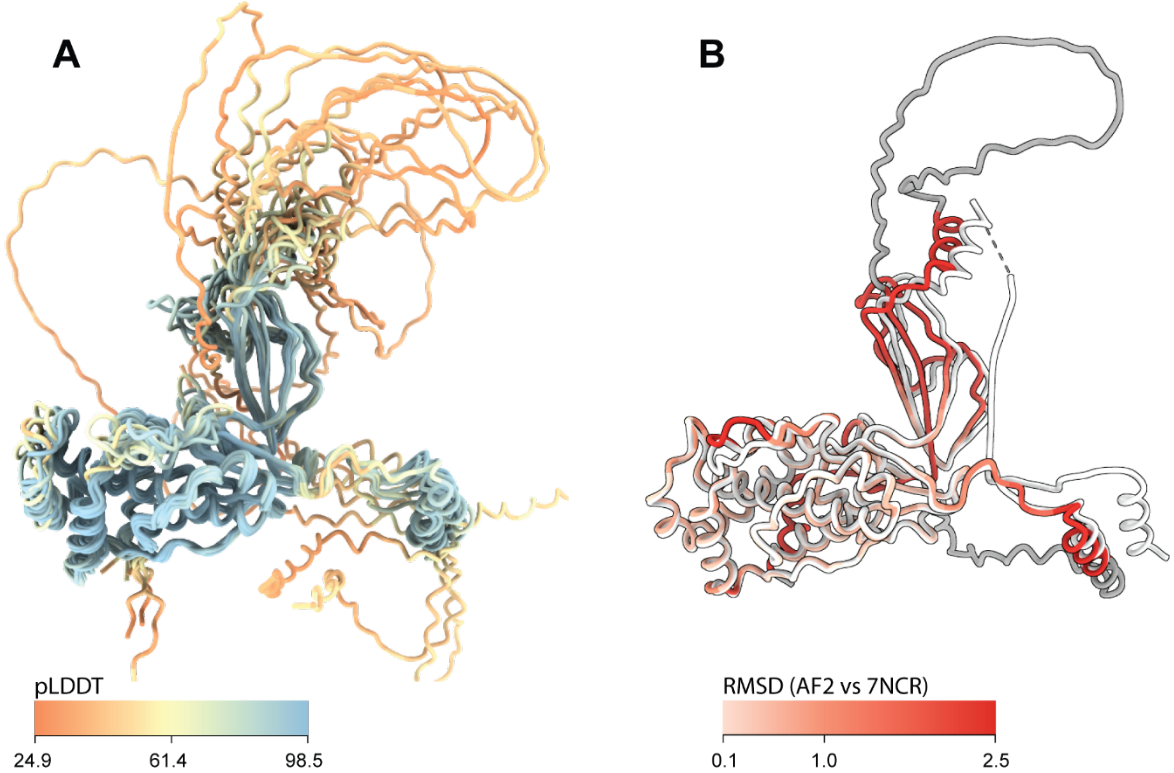

Figure S10. ***Deltapartitivirus*** **(long form CP) structural alignment.**

(A) The predicted CP sequences of long form deltapartitiviruses were aligned using the matchmaker command in ChimeraX version 1.6 using pepper cryptic virus 1 as the reference. The structures include those predicted by alphafold2 (Table S5 below) and are coloured by the predicted local distance difference test (pLDDT) scores. **(B)** Alignment of the alphafold2 prediction and experimentally determined PCV-1 CP structure (PDB 7NCR), coloured by root mean square deviation.

Table S5. ***Deltapartitivirus*** **(long form CP) sequences included in this study.**

Accession	Virus name (genome segment for tripartite viruses	Length (aa)	pLDDT[a]
CDM74096.1	Arhar cryptic virus-II[b]	389	77.74
YP_009508069.1	Beet cryptic virus 2 (RN2b)	393	82.87
ATJ00053.1	Medicago sativa deltapartitivirus 1[b]	394	79.56
YP_009362098.1	Carnation cryptic virus 3[b]	411	83.35
YP_009466860.1	Pepper cryptic virus 1	412	81.37
YP_006390090.1	Persimmon cryptic virus[b]	415	81.69
QED42861.1	Amaranthus cryptic virus 2[b]	421	83.18
YP_009508067.1	Beet cryptic virus 2 (RNA2a)	426	81.52
YP_009351848.1	Pepper cryptic virus 2	430	79.22
QED42859.1	Amaranthus cryptic virus 1[b]	432	79.39
QBC66122.1	Cucumis melo cryptic virus[b]	480	74.21

a - global score for model 1 in all cases
b - not formally assigned to deltapartitiviruses

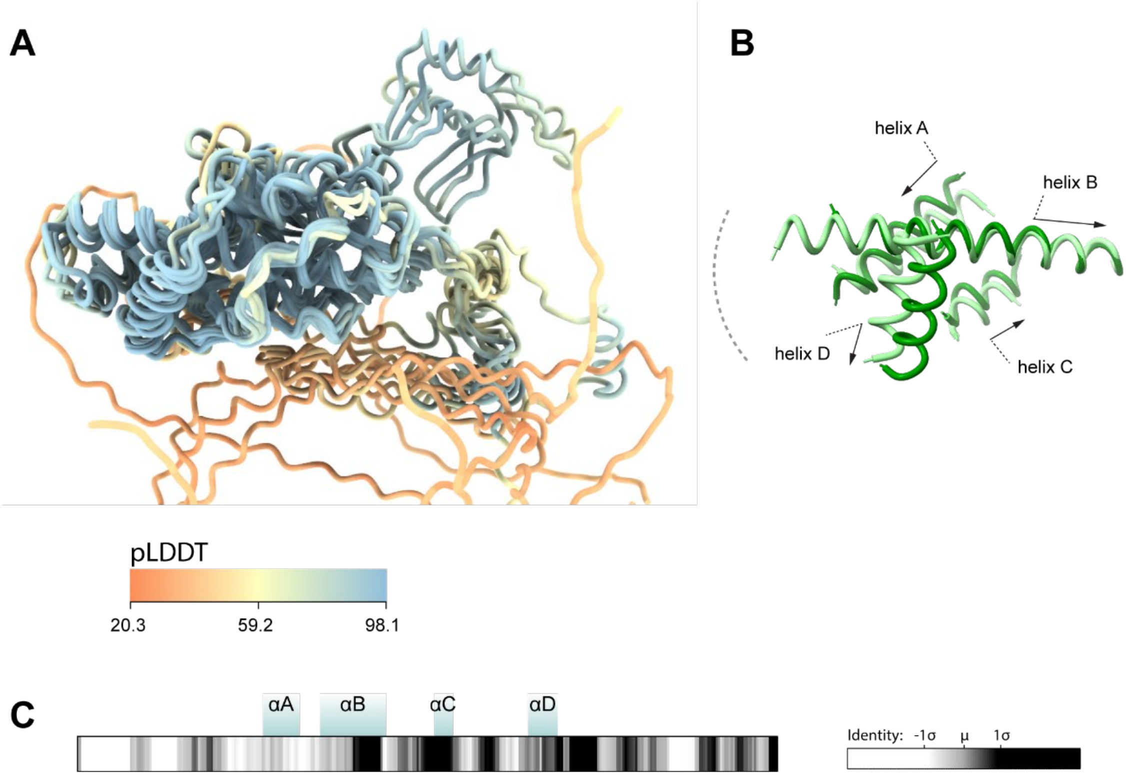

Figure S11. Structural analysis of short form CP deltapartitiviruses.

(A) The predicted CP sequences of short form deltapartitiviruses were aligned using the matchmaker command in ChimeraX version 1.6 using fig cryptic virus as the reference. The structures include those predicted by alphafold2 (Table S6 below) and are coloured by the predicted local distance difference test (pLDDT) scores. **(B)** Alignment of the core helices of the short form (light green) and long form (dark green) deltapartitiviruses, annotated with the direction of the helices. **(C)** Position of the core helices within the primary structure of the short form deltapartitiviruses with the sequence identity shown as a grey-scale heat map scaled to the mean identity calculated over a 10-residue sliding window.

Table S6. Short form *Deltapartitivirus* CP sequences included in this study.

Accession	Virus name (genome segment for tripartite viruses)	Length (aa)	pLDDT[a]
ARO72611.1	Spinach deltapartitivirus 1[b]	335	87.21
YP_004429259.1	Fig cryptic virus	337	89.65
QBQ83755.1	Camellia oleifera cryptic virus 1[b]	344	77.33
YP_001274392.1	Fragaria chiloensis cryptic virus[b] (RNA3)	346	79.22
YP_001686784.1	Raphanus sativus cryptic virus 2[b] (RNA2)	346	75.48
YP_001686788.1	Rose cryptic virus 1[b] (RNA3)	346	80.25
YP_001686785.1	Raphanus sativus cryptic virus 2[b] (RNA3)	347	76.61
YP_001274393.1	Fragaria chiloensis cryptic virus[b] (RNA2)	348	77.20
YP_001686787.1	Rose cryptic virus 1[b] (RNA2)	348	76.54

a - global score for model 1 in all cases

b - not formally assigned to deltapartitiviruses

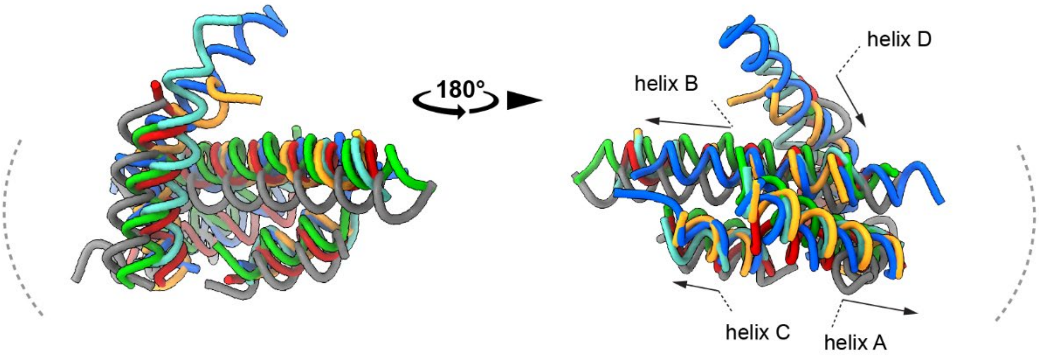

Figure S12. Conservation of core helices including human picobirnavirus CP.

Alignment of the core helices of representative CP structures from each recognised partitivirus genus and human picobirnavirus, annotated with the direction of the helix. The colour of the conserved helical core corresponds to the individual genus members shown in figures 4 and 5 of the manuscript: alphapartitivirus (AF2 - YP_086755) in red; betapartitivirus (PDB - 8PHH) in cyan; gammapartitivirus (PDB - 3ES5) in blue; cryspovirus (AF2 – YP_009508066) in yellow; deltapartitivirus (PDB - 7NCR) in green, picobirnavirus (6Z8D) in black.

References

1. Minh BQ, Schmidt HA, Chernomor O, Schrempf D, Woodhams MD, von Haeseler A, et al. IQ-TREE 2: New Models and Efficient Methods for Phylogenetic Inference in the Genomic Era. Molecular Biology and Evolution. 2020;37(5):1530-4.
2. Minh BQ, Dang CC, Vinh LS, Lanfear R. QMaker: Fast and Accurate Method to Estimate Empirical Models of Protein Evolution. Systematic Biology. 2021;70(5):1046-60.
3. Hoang DT, Chernomor O, von Haeseler A, Minh BQ, Vinh LS. UFBoot2: Improving the Ultrafast Bootstrap Approximation. Molecular Biology and Evolution. 2018;35(2):518-22.
